# Supplementary material for: Cellulose Nanocrystal-Based Emulsion of Thyme Essential Oil: Preparation and Characterisation as Sustainable Crop Protection Tool
Source: Molecules. 2023 Nov 30;28(23):7884. doi: 10.3390/molecules28237884 (PMC10707935; doi:10.3390/molecules28237884)
Supplement: Supplementary file 1 [file molecules-28-07884-s001.zip › molecules-2737247-supplementary.pdf]

Cellulose nanocrystals-based emulsion of thyme essential oil: preparation and characterization as sustainable crop protection tool

Francesca Baldassarre [1, 2] *, Daniele. Schiavi [3], Veronica Di Lorenzo [3], Francesca Biondo [1], Viviana Vergaro [1, 2], Gianpiero Colangelo [4], Giorgio Mariano Balestra [3] and Giuseppe Ciccarella[1, 2] *

[1] Department of Biological and Environmental Sciences, UdR INSTM of Lecce University of Salento, Via Monteroni, 73100 Lecce, Italy

[2] Institute of Nanotechnology, CNR NANOTEC, Consiglio Nazionale delle Ricerche, Via Monteroni, 73100 Lecce, Italy

[3] Department of Agriculture and Forest Sciences (DAFNE), University of Tuscia, Via S. Camillo de Lellis, snc, 01100 Viterbo, Italy

[4] Department of Engineering for Innovation, University of Salento, Via Monteroni, 73100 Lecce, Italy

Supplementary Material

Summary

Supplementary figure 1 UV-vis spectra of Th-EO in cream layer and remaining emulsion volume of CNCs@Th-EO NE_2 formulation after 30 days of storage at room temperature.

Supplementary table 1 TPC data of nanoemulsions after storage at room temperature and at 4°C.

Supplementary table 2 DLS parameters of CNCs@Th-EO NE_1 formulation over time.

Supplementary figure 2 SEM image of used CNCs

Supplementary figure 3 TEM images of Th-EO/surfactant micelles in CNCs@Th-EO NE_1 formulation at different magnification; B is a zoom image of A and D is a zoom image of C.

Supplementary figure 4 Δ Back Scattering % *versus* height (mm) plot of CNCs@Th-EO NE_1 formulation at T 0 and T 30 days, following Turbiscan LabExpert scanning every 2 hours for 24h.

Supplementary figure 1

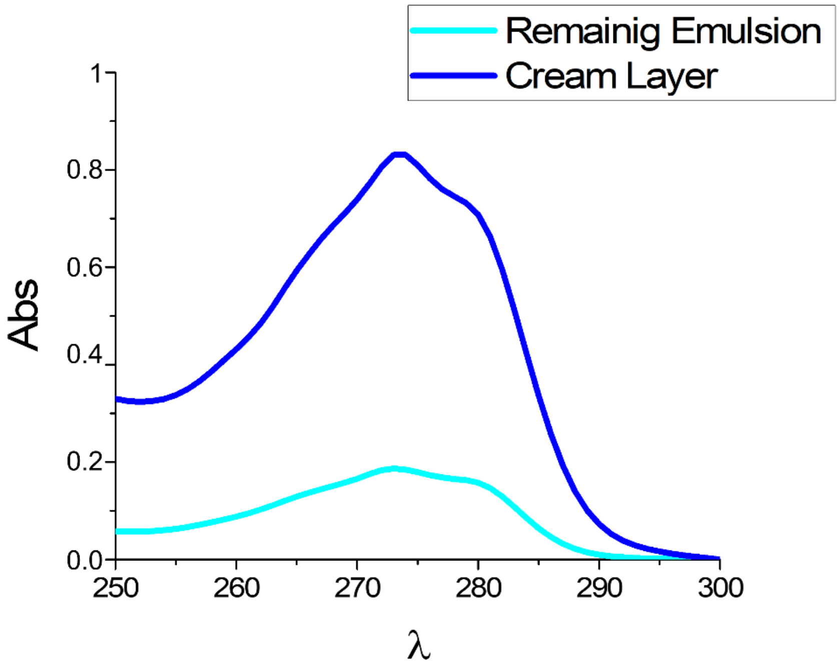

Figure S1. UV-vis spectra of Th-EO in cream layer and in remaining emulsion volume of CNCs@Th-EO NE_2 formulation after 30 days of storage at room temperature.

Supplementary table 1

Sample	μg GAE/mg OET (RT)	μg GAE/mg OET (4°C)
CNCs@Th-EO NE_1	236±12	228±17
CNCs@Th-EO NE_3	218±31	226±14
CNCs@Th-EO NE_4	226±14	224±17

Table S1. TPC data of nanoemulsions after storage at room temperature and at 4°C. TPC assay was made at the same EO concentration of 20 μg/ml that was determined by UV-vis spectrometric analysis.

Supplementary table 2

Time (days)	Z-average diameter (nm)	PdI	ζ-potential
0 *	227±2	0,26±0.012	-26±0.35
7	230±4	0,23±0.007	-27,1±0.2
14	227±4	0,23±0.005	-24,5±0.3

Table S2 .DLS parameters of CNCs@Th-EO NE_1 formulation after preparation (*following solvent evaporation), after 7 and 14 days of storage at RT.

Supplementary figure 2

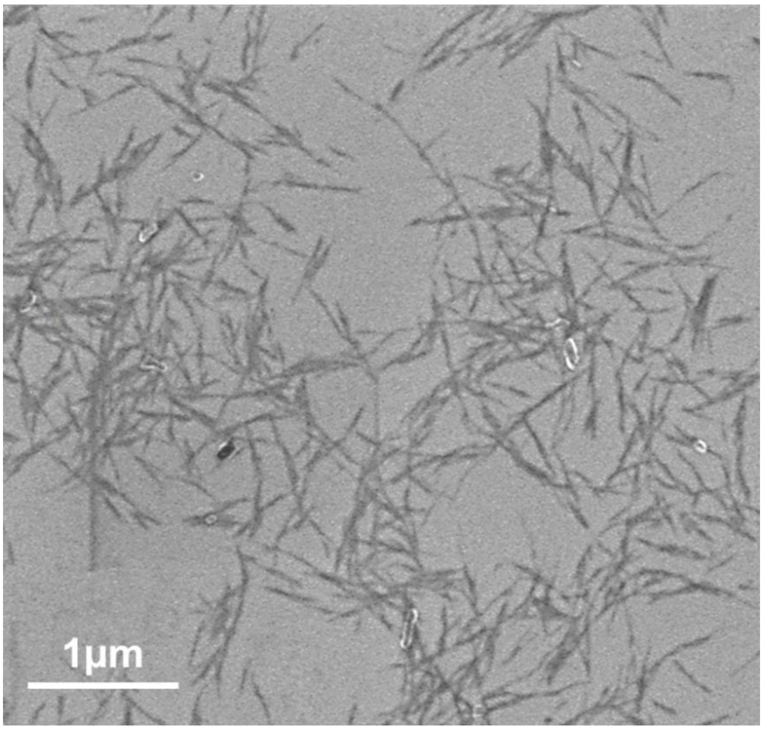

Figure S2. EM image of CNCs in 6% w/v aqueous gel from CelluloseLab.

Supplementary figure 3

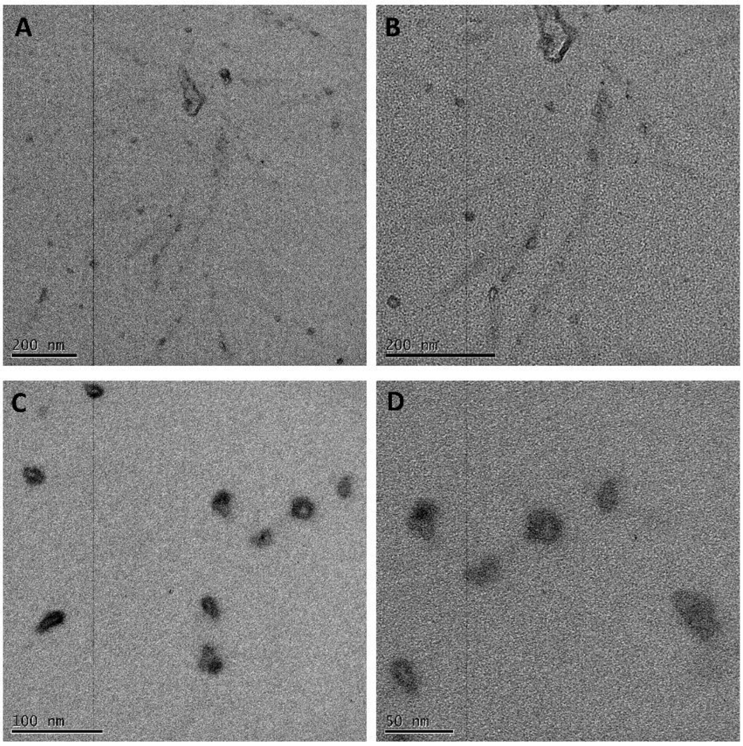

Figure S3. TEM images of Th-EO/surfactant micelles in CNCs@Th-EO NE_1 formulation at different magnification; B is a zoom image of A and D is a zoom image of C. 1 A-B images showed also the presence of CNCs.

Supplementary figure 4

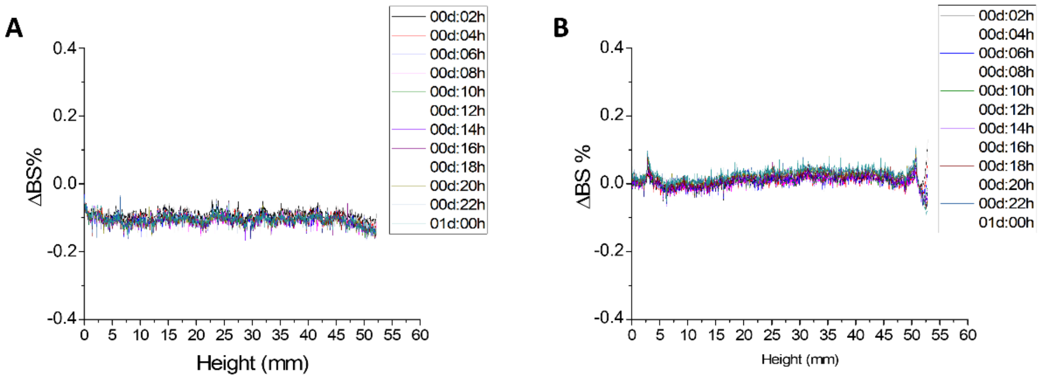

Figure S4. ΔBS% *versus* height (mm) plot of CNCs@Th-EO NE_1 formulation at T 0 (A) and T 30 days (B), following Turbiscan LabExpert scanning every 2 hours for 24h.
